# Endolymphatic Na^+^ and K^+^ Concentrations during Cochlear Growth and Enlargement in Mice Lacking *Slc26a4*/pendrin

**DOI:** 10.1371/journal.pone.0065977

**Published:** 2013-05-31

**Authors:** Xiangming Li, Fei Zhou, Daniel C. Marcus, Philine Wangemann

**Affiliations:** Anatomy and Physiology Department, Kansas State University, Manhattan, Kansas, United States of America; University of South Florida, United States of America

## Abstract

*Slc26a4* ^Δ/Δ^ mice are deaf, develop an enlarged membranous labyrinth, and thereby largely resemble the human phenotype where mutations of *SLC26A4* cause an enlarged vestibular aqueduct and sensorineural hearing loss. The enlargement is likely caused by abnormal ion and fluid transport during the time of embryonic development, however, neither the mechanisms of ion transport nor the ionic composition of the luminal fluid during this time of development are known. Here we determine the ionic composition of inner ear fluids at the time at which the enlargement develops and the onset of expression of selected ion transporters. Concentrations of Na^+^ and K^+^ were measured with double-barreled ion-selective electrodes in the cochlea and the endolymphatic sac of *Slc26a4* ^Δ/+^, which develop normal hearing, and of *Slc26a4* ^Δ/Δ^ mice, which fail to develop hearing. The expression of specific ion transporters was examined by quantitative RT-PCR and immunohistochemistry. High Na^+^ (∼141 mM) and low K^+^ concentrations (∼11 mM) were found at embryonic day (E) 16.5 in cochlear endolymph of *Slc26a4* ^Δ/+^ and *Slc26a4* ^Δ/Δ^ mice. Shortly before birth the K^+^ concentration began to rise. Immediately after birth (postnatal day 0), the Na^+^ and K^+^ concentrations in cochlear endolymph were each ∼80 mM. In *Slc26a4* ^Δ/Δ^ mice, the rise in the K^+^ concentration occurred with a ∼3 day delay. K^+^ concentrations were also found to be low (∼15 mM) in the embryonic endolymphatic sac. The onset of expression of the K^+^ channel KCNQ1 and the Na^+^/2Cl^−^/K^+^ cotransporter SLC12A2 occurred in the cochlea at E19.5 in *Slc26a4* ^Δ/+^ and *Slc26a4* ^*Δ/Δ*^ mice. These data demonstrate that endolymph, at the time at which the enlargement develops, is a Na^+^-rich fluid, which transitions into a K^+^-rich fluid before birth. The data suggest that the endolymphatic enlargement caused by a loss of *Slc26a4* is a consequence of disrupted Na^+^ transport.

## Introduction

Enlargement of vestibular aqueduct (EVA) is a common inner ear malformation found in children with non-syndromic sensorineural hearing loss and hearing loss associated with Pendred syndrome [1]. Mutations of *SLC26A4* are a prevalent cause of EVA and of progressive and often fluctuating hearing loss with an onset before or around speech acquisition [2], [3], [4], [5], [6]. *SLC26A4* codes for pendrin, an anion exchanger that functions as a Cl^−^/HCO_3_^−^ exchanger in the inner ear [7]. In some populations, mutations in *SLC26A4* occur in 13–14% of deaf subjects [8], [9].

*Slc26a4* ^Δ/Δ^ mice closely resemble the human phenotype, since *Slc26a4* ^Δ/Δ^ mice acquire an enlargement of the vestibular aqueduct during embryonic development. The vestibular aqueduct is a bony tunnel that forms around the epithelia-lined endolymphatic duct. Swelling of the fluid-filled epithelium of the inner ear in *Slc26a4* ^Δ/Δ^ mice leads to an enlargement of the vestibular aqueduct. *Slc26a4* ^Δ/Δ^ mice fail to acquire hearing during the postnatal phase of development [7], [10]. Studies on *Slc26a4* ^Δ/Δ^ mice suggest that the endolymphatic enlargement entails a ∼10-fold larger luminal volume in the cochlea, that the endolymphatic acidification entails a ∼2-fold higher H^+^ ion concentration, and that the enlargement and acidification are key events that mark the onset of the pathology [11], [12].

The development of the murine inner ear begins with an ectodermal invagination that forms an otocyst at embryonic day (E) 9.5. The otocyst is filled with amniotic fluid, which is a NaCl-rich fluid [13]. The otocyst extends two protrusions: one to form scala media of the cochlea and the other to form the endolymphatic duct and sac, while the middle of the otocyst is reorganized into the vestibular labyrinth [14]. The lumen of the endolymphatic sac opens at E10.5 and the lumen of the cochlea begins to open at E14.5. The fluid inside the lumen is called endolymph and the fluid surrounding the epithelium is called perilymph. In *Slc26a4* ^Δ/Δ^ mice, the enlargement of the endolymphatic lumen begins at E14.5, which coincides with cochlear growth and lumen formation between E14.5 and E18.5 [1], [15].

**Figure 1:**
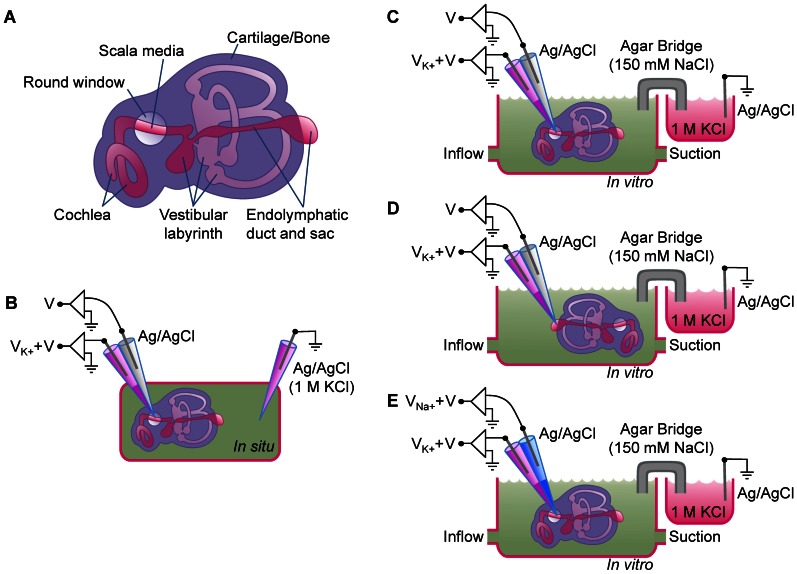
Schematic diagrams of the inner ear and of experimental configurations. A) Diagram of the bone-enclosed inner ear consisting of the cochlea, the vestibular labyrinth and the endolymphatic duct and sac. Note, that scala media of the cochlea is accessible via the round window and that the endolymphatic sac is the only structure not enclosed in bone. B–E) Experimental configurations for the measurement of voltages and of Na+ and K+ concentrations. Voltage electrodes were used to record the voltage (V) and ion-selective electrodes, which were filled at the tip with liquid ion exchanger, recorded a signal that consisted of the sum of the voltage (V) and the ion-voltage (VK+ or VNa+). B) Configuration for in situ measurements of voltage and K+ concentrations in the cochlea. C–E) Configuration for in vitro measurements. Temporal bones were isolated and superfused in a bath chamber that was outfitted with an inflow and a suction outlet. C) Configuration for in vitro measurements of voltage and K+ concentrations in the cochlea. D) Configuration for in vitro measurements of voltage and K+ concentrations in the endolymphatic sac. E) Configuration for in vitro measurements of Na+ and K+ concentrations in the cochlea.

The mesenchymal tissue surrounding the cochlea, the vestibular labyrinth and the endolymphatic duct forms cartilage during embryonic development that is later converted into bone (Fig. 1A). In addition, the cochlea and the vestibular labyrinth are enclosed in the temporal bone. Access to scala media of the cochlea is available via the round window that is located near the base of the cochlea. The endolymphatic sac is the only part of the inner ear that is not enclosed in cartilage or bone.

The mechanisms that lead to cochlear enlargement in *Slc26a4* ^*Δ/Δ*^ mice are not known. It is conceivable that fluid accumulation leading to the enlargement occurs in the wake of an abnormally low rate of ion absorption or an abnormally high rate of ion secretion. Ion transport in the inner ear has so far mainly been studied in adult animals, when endolymph consists of a K^+^-rich fluid [16]. The enlargement of the endolymphatic lumen in *Slc26a4* ^*Δ/Δ*^ mice, however, occurs during embryonic development. Thus, it is of great interest to determine whether endolymph in *Slc26a4* ^*Δ/Δ*^ and *Slc26a4* ^Δ/+^ mice at the time of enlargement is a K^+^-rich fluid, as observed in the adult inner ear, or whether endolymph at this time is a Na^+^-rich fluid similar in composition to amniotic fluid. The determination of the ion composition during the phase of enlargement is the very first step for understanding how the enlargement occurs. Thus the main goal of the present study was to determine the Na^+^ and K^+^ concentrations in cochlear endolymph during embryonic development.

## Methods

### Ethics Statement

All procedures involving animals were approved by the Institutional Animal Care and Use Committee at Kansas State University (IACUC#: 2961, 2925 and 3245).

### Animals

A colony of *Slc26a4* ^Δ/+^ and *Slc26a4* ^Δ/Δ^ mice was maintained at Kansas State University. *Slc26a4* ^Δ/+^ dams and *Slc26a4* ^Δ/Δ^ sires were housed in monogamous pairs. Litter sizes averaged 4.3 pups with *Slc26a4* ^Δ/+^ and *Slc26a4* ^Δ/Δ^ offspring in the near Mendelian ratio of 50.1 to 49.9. The average gestational period was 21 days. Gestational age was counted from the day when a vaginal plug was detected. This day was set to embryonic (E) day 0.5 (E0.5). Pregnancies were verified by ultrasound (Terason t3000, Universal Medical Systems, Bedford Hills, NY). The colony was maintained free of known and suspected murine pathogens, as reported earlier [15]. *Slc26a4* ^Δ/+^ and *Slc26a4* ^Δ/Δ^ mice ranging in age from E14.5 to P72 were used in the present study. Time-pregnant dams were deeply anesthetized with 4% tri-bromo-ethanol and sacrificed by decapitation after harvesting embryos by sterile laparotomy. Embryos were sacrificed by decapitation. Neonatal mice (P0) were anesthetized by a combination of intraperitoneal (i.p.) injection of 0.014 ml/g body weight of 4% tri-bromo-ethanol and rapid cooling on a slush of ice. Older mice (>P3) were anesthetized solely by i.p. injection of 0.014 ml/g body weight of 4% tri-bromo-ethanol. Neonatal and older mice were sacrificed by decapitation under deep anesthesia.

### Measurement of voltage, Na^+^ and K^+^ concentrations

Voltage, Na^+^ and K^+^ concentrations were measured with double-barreled ion-selective electrodes. Measurements in prenatal and neonatal mice were carried out using a novel *in vitro* approach. This novel approach was developed since the well-established *in situ* approach is not suitable for measurements in prenatal mice. Limitations of the *in vitro* approach were established by comparing results from postnatal mice obtained *in vitro* to data obtained *in situ*.

For *in situ* measurements of K^+^ concentrations, one barrel of the double-barreled electrode was used to measure the K^+^ concentration and the other barrel was used to measure the transepithelial voltage (Fig. 1B). Electrodes were manufactured using established protocols [17]. Briefly, the K^+^ selective barrel was silanized prior to being filled at the tip with liquid ion exchanger (Ionophore I - cocktail B, Cat# 60398, Sigma-Aldrich) and backfilled with 1 M KCl solution. The voltage barrel was filled with 500 mM NaCl solution. Each barrel was connected via a Ag/AgCl electrode to a dual electrometer with high input-resistance (FD223, World Precision Instruments, Sarasota, FL).

Mice between ages P62 and P72 were deeply anesthetized and maintained at 37°C body temperature using a heated platform. Surgery consisted of a tracheotomy to maintain unobstructed ventilation, the subdermal installation of a flowing 1 M KCl reference electrode in the chest region, and of a ventral approach to the temporal bone, using established methods [17]. Briefly, a small opening in the temporal bone was made and measurements were obtained by guiding the electrode through the round window into the perilymph-filled scala tympani and then into the endolymph-filled scala media. Anoxia was induced by i.p. injection of succinylcholine chloride (1 mg/g body weight) after obtaining stable readings of the K^+^ concentration and the endocochlear potential.

For *in vitro* measurements of K^+^ and Na^+^ concentrations, temporal bones were isolated from pre- and postnatal mice and placed into a bath chamber superfused with warm (37°C) artificial perilymph (in-line heater SHM-8 and controller TC-344B, Warner instruments, Hamden, CT). Artificial perilymph contained (in mM): 135 NaCl, 25 NaHCO_3_, 4 KCl, 1.5 CaCl_2_, 1 MgCl_2_ and 5 glucose and was bubbled with a humidified gas mixture consisting of 5% CO_2_ and 95% O_2_ to set the pH to pH 7.3 – pH 7.4. The bath chamber was grounded via a Ag/AgCl electrode that was bathed in a vial filled with 1 M KCl solution and connected to the bath chamber via an agar-bridge made from 150 mM NaCl solution (Fig. 1C-E). For measurements in the cochlea, an approach via the round-window was used. For *in vitro* measurements of K^+^ concentrations, one barrel of the double-barreled electrode was used to measure the K^+^ concentration and the other barrel was used to measure the transepithelial voltage (Fig. 1C). For *in vitro* measurement of Na^+^ concentrations, one barrel of the double-barreled electrode was used to measure the Na^+^ concentration and the other barrel was used to measure the K^+^ concentration as an indicator for successful penetration into the endolymph-filled scala media (Fig. 1E). This unusual configuration was found necessary since at the ages of interest the transepithelial voltage was found to be near zero and the Na^+^ concentration in endolymph was found to be similar to the concentration in perilymph such that neither the recording of the voltage nor the recording of the Na^+^ concentration could serve as an indicator for successful penetration into the endolymph-filled scala media.

Dual ion selective electrodes were manufactured using a modified protocol. Both barrels were silanized by a 20 s exposure to silane vapors generated by injecting 10 µl di-methyl-di-chloro-silane into a 150 ml beaker sitting on a hot-plate heated to 210°C. Silanized electrodes were baked for 2 hr at 180°C. Tips were broken to an outer diameter of 10–12 µm. The Na^+^ electrodes were filled at the tip with a calixarene-based liquid ion exchanger (Cocktail 2 consisting of (%w/w) 3.5% Na^+^ ionophore X (Cat# 71747, Sigma-Aldrich), 95.9% 2-nitrophenyl-octyl-ether (Cat# 73732, Sigma-Aldrich), 0.6% potassium-tetrakis-(4-chlorophenyl)-borate (Cat# 60591, Sigma-Aldrich) and backfilled of 1 M NaCl [18]. Electrodes were stored in 500 mM KCl solution for 2-4 hr prior to experiments.

Calibration of ion selective electrodes in warm (37°C) solutions was performed immediately after withdrawal of the electrodes from the temporal bone. All measurements were corrected for calculated liquid junction potentials (JPCalc in Clampex, Molecular Devices, Silicon Valley, CA [19]).

Data were recorded via a flat-bed chart recorder (BD12E, Kipp & Zonen, The Netherlands) for easy annotation and also were recorded digitally (Digidata 1322A and AxoScope 9, Molecular Devices) for easy analysis using custom software written by P.W. (Origin 6.0, The Origin Company, Northhampton, MA).

### Quantitative RT-PCR

Total RNA was isolated from the cochlea of *Slc26a4* ^Δ/+^ and *Slc26a4* ^Δ/Δ^ mice between E14.5 and P8 (RNeasy micro, Qiagen, Valencia, CA). Quantity and quality of total RNA were evaluated by microfluidic electrophoresis (BioAnalyzer, Agilent, Santa Clara, CA) and by microliter absorption photometry (Nanodrop, Wilmington, DE). RNA samples were accepted only when they were free of contamination and when RIN was >7.0, which indicates excellent quality. RIN is a quality indicator on a scale from 0 (worst) to 10 (best) that is computed from microfluidic electrophoresis runs (BioAnalyzer, Agilent).

Total RNA, primers, enzymes and buffers necessary for quantitative RT-PCR reactions were assembled with the assistance of an automatic pipetting station (Biomek NX^P^, Beckman Coulter, Fullerton, CA) with hardware modifications and software programming by P.W. Reactions were carried out in 96-well plates with each well containing ∼10 ng of total RNA, gene specific primers, SYBR-green, and an enzyme mix containing reverse transcriptase and DNA polymerase (iScript, BioRad, Hercules, CA) in a total volume of 25 µl. Reverse transcription was performed for 10 min at 50°C and terminated by heating to 95°C for 5 min (OneStepPlus, Applied Biosystems, Foster City, CA). PCR consisted of 40 cycles of 10 s melting at 95°C, 30 s annealing and elongation at 58°C, and 15 s fluorescence detection at 78°C (OneStepPlus, Applied Biosystems).

Gene specific primers were designed using software (Primer 3.0 [20]). Primer pairs spanned introns to discourage amplification of genomic DNA (Table 1). Amplification of a single product of the appropriate size was verified by microfluidic electrophoresis (BioAnalyzer, Agilent).

**Table 1:** Primers.

Gene	Genbank	Genbank	Genbank	Location
*18S*	NR_003278	Left: gag gtt cga aga cga tca ga	316 bp	
		Right: tcg ctc cac caa cta aga ac		
*Kcnq1*	NM_008434	Left: ttc tcc tcc tac ttt gtc tac ttg g	262 bp	Exon 6
		Right: tct gcc tct gct tct gct		Exon 8
*Slc12a2*	NM_009194	Left: gga agc aaa ggc tca gat tg	345 bp	Exon 7–8
		Right: aca aca cac gaa ccc aca ga		Exon 8–9
*Atp1a1*	NM_144900	Left: tgc ccg cct caa cat tcc	291 bp	Exon 14
		Right: gac aca tca gag cca aca atc c		Exon 16
*Scnn1a*	NM_011324	Left: agg aag gac tgg aaa atc g	248 bp	Exon 2
		Right: atg ggg tgg tgg aac tga		Exon 4
*Scnn1b*	NM_011325	Left: ctt cac gcc tat ctt cta ccc	268 bp	Exon 5
		Right: gtc cac cag cac ccc aat		Exon 7
*Scnn1g*	NM_011326	Left: ggt ggg att tca gtt gtg ct	282 bp	Exon 3–4
		Right: agg atg gtg gcg ttt tct ct		Exon 5

The number of template molecules (*cDNA_templates_*) was estimated according to 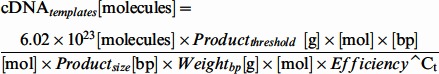



where 6.02×10^23^ molecules/mol represents Avogadro's number, *Product_threshold_* is the weight of the PCR-product at threshold (0.49×10^−9^ g) that was obtained from calibration experiments, *Product_size_* is the size of the product in base pairs (bp), *Weight_bp_* is average weight of one bp (660 g/mol), *Efficiency* is the PCR-efficiency obtained from the slope of the log-linear phase of the growth curve [21] and *C_t_* is the cycle at which the fluorescence of the product molecules reaches a common threshold chosen in the middle of the log-linear part of the growth curve.

### Confocal immunohistochemistry

Temporal bones were isolated from *Slc26a4* ^Δ/+^ and *Slc26a4* ^Δ/Δ^ mice and fixed at 4°C in PBS-solution to which 4% paraformaldehyde (Cat# 15714, Electron Microscopy Sciences, Hatfield, PA) had been added. PBS-solution contained (mM): 137 NaCl, 2.7 KCl, 10.1 Na_2_HPO_4_, and 1.8 KH_2_PO_4_, pH 7.4. Fixed tissues were processed through a sucrose gradient (10% and 20%, each 30 min, followed by 30% overnight, all solutions in PBS at 4°C), infiltrated with polyethylene glycol (Cat# 72592-B, Electron Microscopy Sciences) and cryo-sectioned (12 µm, CM3050S, Leica, Germany). Mid-modiolar sections of the cochlea were mounted on charged slides (Cat# 22-230-900, Fisher) and blocked for 1 hr with 5% bovine serum albumin (BSA) in PBS-solution containing 0.2% TritonX-100 (PBS-TX solution).

Sections were incubated at 4°C overnight with primary antibodies, goat anti-KCNQ1 (1∶200, Cat# SC-10646, Santa Cruz, Dallas, TX) or rabbit anti-SLC12A2 (1∶200, Cat# AB3560P, Chemicon, Temecula, CA). Sections were washed 3 times for 1 min each with PBS-TX solution and incubated at room temperature for 1 hr with diluted secondary antibodies, Alexa Fluor 594 chicken-anti-goat (1∶1000, Cat# A21468, Invitrogen, Grand Island, NY) or Alexa Fluor 594 goat-anti-rabbit (1∶1000, Cat# A11037, Invitrogen). Primary and secondary antibodies were diluted in PBS-TX solution containing 2.5% BSA. After washing three times in PBS-TX, sections were stained with phalloidin 488 (1∶40, Cat# A-12379, Invitrogen) and DAPI (1∶1000; Cat# D-3571, Invitrogen). Stains were diluted in PBS-TX solution without BSA. After staining, sections were washed another 3 times in PBS-TX solution and cover-slipped with mounting medium (Cat# H-1400, Vector laboratories, Burlingame, CA) and examined by confocal laser scanning microscopy (LSM 510 Meta, Carl Zeiss, Göttingen, Germany).

### Statistics

Numerical data were presented as average ± sem with n being the number of replicates. Statistical significance was determined based on unpaired t-tests. Significance was assumed at p<0.05.

## Results

### Selectivity and sensitivity of K^+^- and Na^+^-selective electrodes

**Figure 2:**
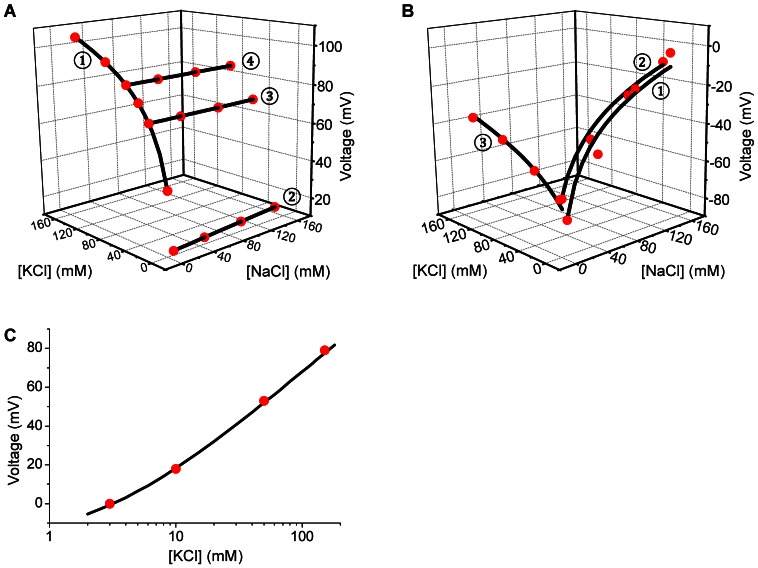
Selectivity and sensitivity of Na^+^ and K^+^-selective electrodes. Electrodes were calibrated using mixtures of NaCl and KCl and the resulting two- or three-dimensional data (*red dots*) were fitted to the Nicolski equation (*black lines*). **A**) Three-dimensional calibration of K^+^ selective electrodes. *Curve 1*: The KCl concentration was varied between 10 and 150 mM in the presence of a constant NaCl concentration of 10 mM. *Curve 2:* The NaCl concentration was varied between 10 to 150 mM in the presence of a constant KCl concentration of 3 mM. *Curve 3*: The NaCl concentration was varied between 10 to 150 mM in the presence of a constant KCl concentration of 35 mM. *Curve 4*: The NaCl concentration was varied between 10 to 150 mM in the presence of a constant KCl concentration of 68 mM. Note, that K^+^ selective electrodes were highly selective for K^+^ which is evident from the steep relationship between the Voltage and the KCl concentration (*Curve 1*) and the flat relationships between the voltage and the NaCl concentration (*Curves 2–4*). **B**) Three-dimensional calibration of Na^+^ selective electrodes. *Curve 1*: The NaCl concentration was varied between 10 and 150 mM in the presence of a constant KCl concentration of 3 mM. *Curve 2*: The NaCl concentration was varied between 10 to 150 mM in the presence of a constant KCl concentration of 13 mM. *Curve 3*: The KCl concentration was varied between 10 to 150 mM in the presence of a constant NaCl concentration of 10 mM. Note, that Na^+^ selective electrodes were highly selective for Na^+^ but, although to a lesser degree, also detected K^+^, which is evident from the steep relationships between the Voltage and the NaCl concentration (*Curve 1 and 2*) and the less steep relationship between the voltage and the KCl concentration (*Curve 3*). **C**) Two-dimensional calibration of K^+^ selective electrodes. Calibration procedures were simplified by using solutions in which the sum of NaCl and KCl was maintained constant at 150 mM.

The sensitivity and selectivity of K^+^ electrodes was evaluated by making recordings in solutions containing various concentrations of KCl and NaCl. Recordings were corrected for calculated liquid junction potentials and corrected measurements were subjected to a three-dimensional fit to the Nicolski equation: 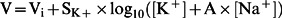



where V represents the measured voltage, V_i_ is an offset term, S_K+_ is the slope representing the sensitivity for K^+^, [K^+^] and [Na^+^] represent the K^+^ and Na^+^ concentrations, and A is the selectivity coefficient. K^+^ electrodes were found to be highly sensitive with S_K+_ near the theoretical value of 60 (52.9±0.5 mV/decade, n = 4) and highly selective with A close the ideal value of zero (0.028±0.005, n = 4). Accordingly, steep voltage changes were recorded when the KCl concentration was changed in the presence of a constant NaCl concentration of 10 mM and nearly no voltage changes were observed when the NaCl concentration was altered at constant KCl concentrations of 3, 35 or 68 mM (Fig. 2A).

The sensitivity and selectivity of Na^+^ electrodes was evaluated in a similar fashion using solutions containing various concentrations of NaCl and KCl. Recorded voltages were corrected for calculated liquid junction potentials and corrected voltages were subjected to a three-dimensional fit to the Nicolski equation: 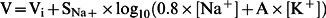



where S_Na+_ is the slope representing the sensitivity for Na^+^ and all other symbols have the same meaning as above. Na^+^ electrodes were found to be highly sensitive with S_Na+_ near the theoretical value of 60 (57.8±0.9 mV/decade, n = 12) and moderately selective with A being different from zero (0.13±0.03, n = 12). Accordingly, steep voltage changes were recorded when the NaCl concentration was changed at constant KCl concentration of 3 or 13 mM and less steep changes were observed when the KCl concentration was altered in the presence of a constant NaCl concentration of 10 mM (Fig. 2B).

### Simplified calibration procedure for K^+^-selective electrodes

The high selectivity of K^+^ electrodes encouraged us to simplify the calibration process for K^+^ electrodes by using solutions composed of KCl and NaCl, where the total cation concentration was kept constant at 150 mM. Recorded voltages were corrected for calculated liquid junction potentials and corrected voltages were subjected to a two-dimensional fit to a simplified Nicolski equation: 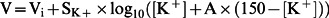



where symbols have the same meaning as above. Again, K^+^ electrodes were found to be highly sensitive with S_K+_ near the theoretical value of 60 (54.9±0.4, n = 137) and highly selective with A near the ideal value of zero (0.018±0.001, n = 137). Accordingly, the relationship between voltage and K^+^ was found to be non-linear (Fig. 2C).

### K^+^ concentration and voltage measurements in situ and in vitro in the cochlea of adult mice

**Figure 3:**
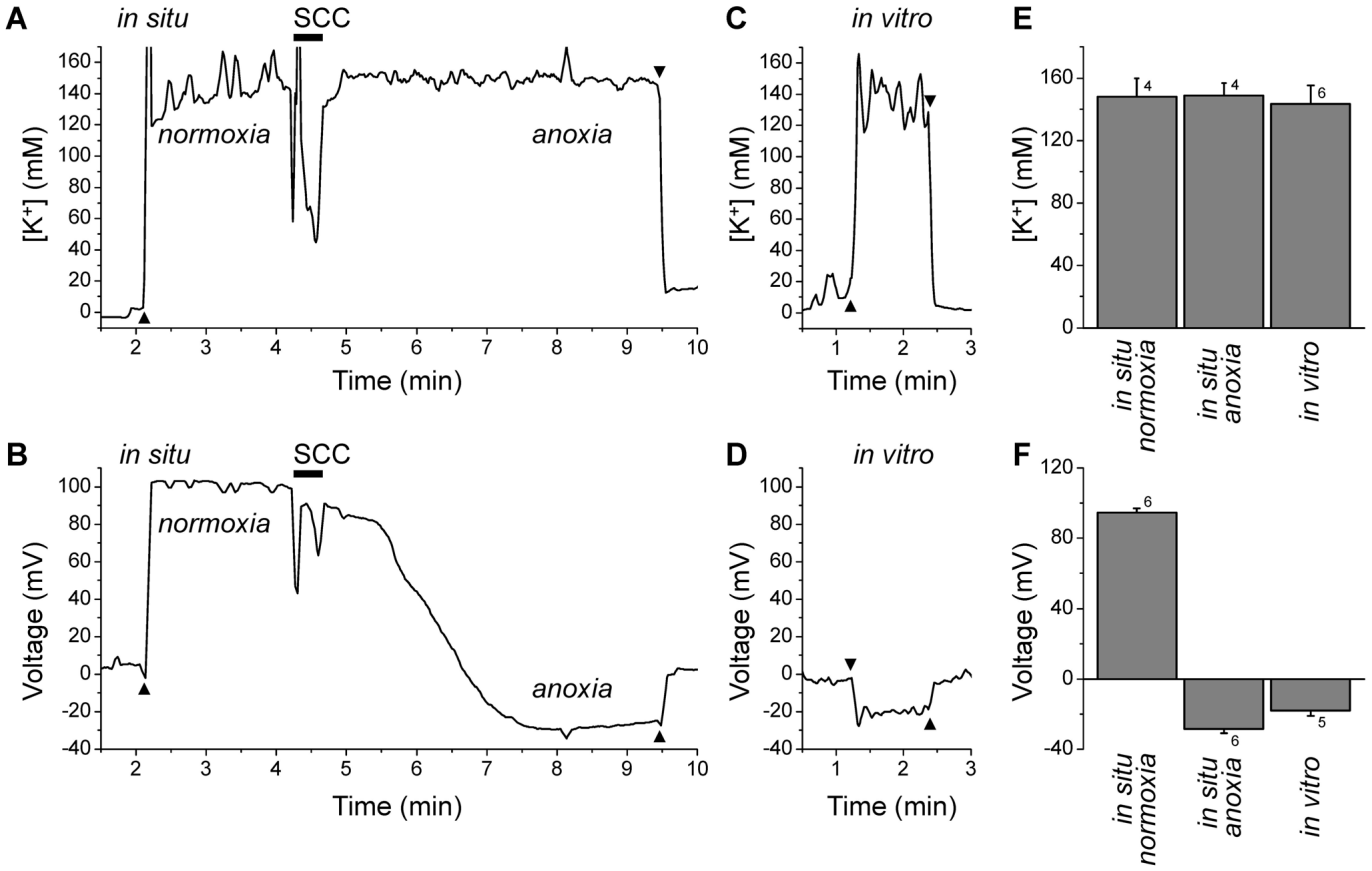
Measurements of the K^+^ concentration and the endocochlear potential *in situ* and *in vitro* in adult *Slc26a4* ^Δ/+^ mice. **A–B**) Representative traces of *in situ* measurements. Penetration and withdrawal from the epithelium are marked (*filled triangles*). Upon establishment of a stable voltage recording, the paralytic agent succinyl-choline chloride (SCC) was injected to induced anoxia. Note that the endocochlear potential, which is positive under normoxic conditions, declined under anoxic conditions within 3 min to negative values. The K^+^ concentration, however, was maintained constant during the recording time. **C–D**) Representative traces of *in vitro* measurements. Penetration and withdrawal from the epithelium are marked (*filled triangles*). **E–F**) Data summary. Note that the K^+^ concentrations recorded *in vitro* were similar to measurements made *in situ* and that the endocochlear potential *in vitro* was negative similar to *in situ* measurements under anoxic conditions. Numbers next to bars represents the number of animals.

Measurements of the endocochlear potential and of ion concentrations in cochlear endolymph are usually performed *in situ* using adult animals, although some investigators have succeeded to obtain measurements in pre-weaning and even neonatal mice [7], [22]. The *in situ* technique, however, cannot be extended to the prenatal phase of development. Therefore, we established an *in vitro* method where measurements of the transepithelial voltage and of endolymphatic ion concentrations are conducted in isolated superfused temporal bones. This method was evaluated by comparing data from adult *Slc26a4* ^Δ/+^ mice (P62-P72) that were obtained with the *in situ* technique and with the *in vitro* method (Fig. 3). Measurements *in situ* yielded an endolymphatic K^+^ concentration of 157±14 (n = 4) mM and an endocochlear potential at normoxic conditions of 95±2 (n = 6) mV. Upon induction of anoxia by injection of succinylcholine, the endocochlear potential declined within 2–3 min to −29±2 (n = 6) mV, while the endolymphatic K^+^ concentration remained stable (153±9 mM, n = 4). Measurements *in vitro* were obtained within 10–15 min after sacrifice and yielded an endolymphatic K^+^ concentration of 144±12 (n = 6) mM, which is similar to results obtained *in situ*, and an endocochlear potential of −16±3 (n = 6) mV, which is similar to the anoxia potential found *in situ*. These data suggest that the *in vitro* method is suitable to evaluate the K^+^ concentration, and presumably the Na^+^ concentration, and that *in vitro* measurements of the transepithelial potential more closely approximate anoxic rather than normoxic conditions.

### K^+^ concentration and voltage measurements in the cochlea and endolymphatic sac

**Figure 4:**
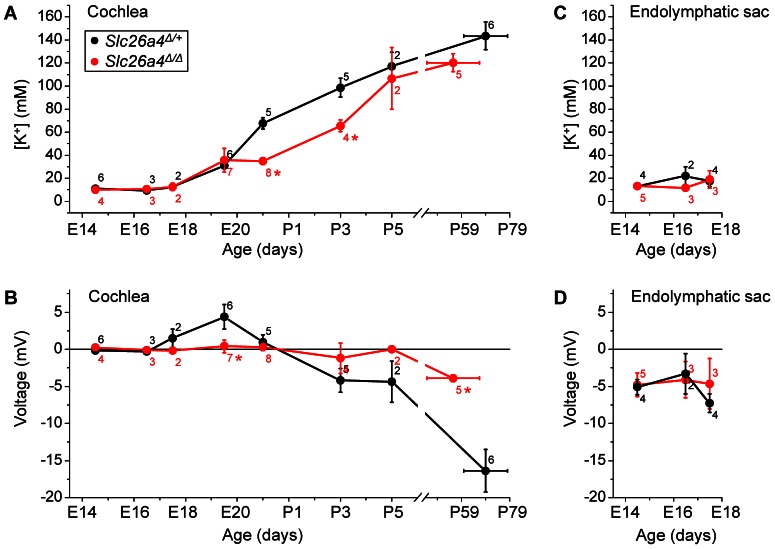
Summary of measurements of the endolymphatic K^+^ concentration and transepithelial voltage *in vitro* in the cochlea and the endolymphatic sac of *Slc26a4* ^Δ/+^ (*black symbols*) and *Slc26a4* ^Δ/Δ^ mice (*red symbols*). **A–B**) Measurements in the cochlea. **C–D**) Measurements in the endolymphatic sac. Measurements in *Slc26a4* ^Δ/Δ^ mice that differed significantly from measurements in *Slc26a4* ^Δ/+^ mice are marked (*).

The *in vitro* method was used to determine the endolymphatic K^+^ concentration and the transepithelial potential in the cochlea and the endolymphatic sac during development. Measurements were made with double-barreled K^+^ selective electrodes in prenatal and neonatal *Slc26a4* ^Δ/+^ and *Slc26a4* ^Δ/Δ^ mice (Fig. 4).

In the cochlea, the endolymphatic K^+^ concentration between age E14.5 and E17.5 was 10.7±0.7 (n = 20) mM with no differences between *Slc26a4* ^Δ/+^ and *Slc26a4* ^Δ/Δ^ mice (Fig. 4A). At E19.5, the endolymphatic K^+^ concentration began to rise in both genotypes. At P0 and P3, the endolymphatic K^+^ concentrations in the cochlea of *Slc26a4* ^Δ/Δ^ mice was significantly lower compared to in *Slc26a4* ^Δ/+^ mice, however, this difference disappeared with further development. The perilymphatic K^+^ concentration was 3.29±0.07 (n = 72) mM and did not change with age and displayed no differences between genotypes. *Slc26a4* ^Δ/+^ mice developed a positive transepithelial voltage of 4.4±1.7 (n = 6) mV at E19.5 (Fig. 4B). With further development, the transepithelial voltage became negative. Transepithelial voltages in *Slc26a4* ^Δ/Δ^ mice remained close to zero till age P5, but was found to be negative in adult mice (−3.9±0.5 mV, n = 5).

In the endolymphatic sac, the endolymphatic K^+^ concentration between ages E14.5 and E17.5 was 15.4±1.6 (n = 21) mM with no differences between *Slc26a4* ^Δ/+^ and *Slc26a4* ^Δ/Δ^ mice (Fig. 4C) and the transepithelial potential was −5.1±0.7 (n = 21) mM, again with no differences between *Slc26a4* ^Δ/+^ and *Slc26a4* ^Δ/Δ^ mice (Fig. 4D). Between ages E14.5 and E17.5, no difference was found between the endolymphatic K^+^ concentration in the cochlea and the endolymphatic sac.

### Na^+^ and K^+^ concentration measurements in the cochlea

**Figure 5:**
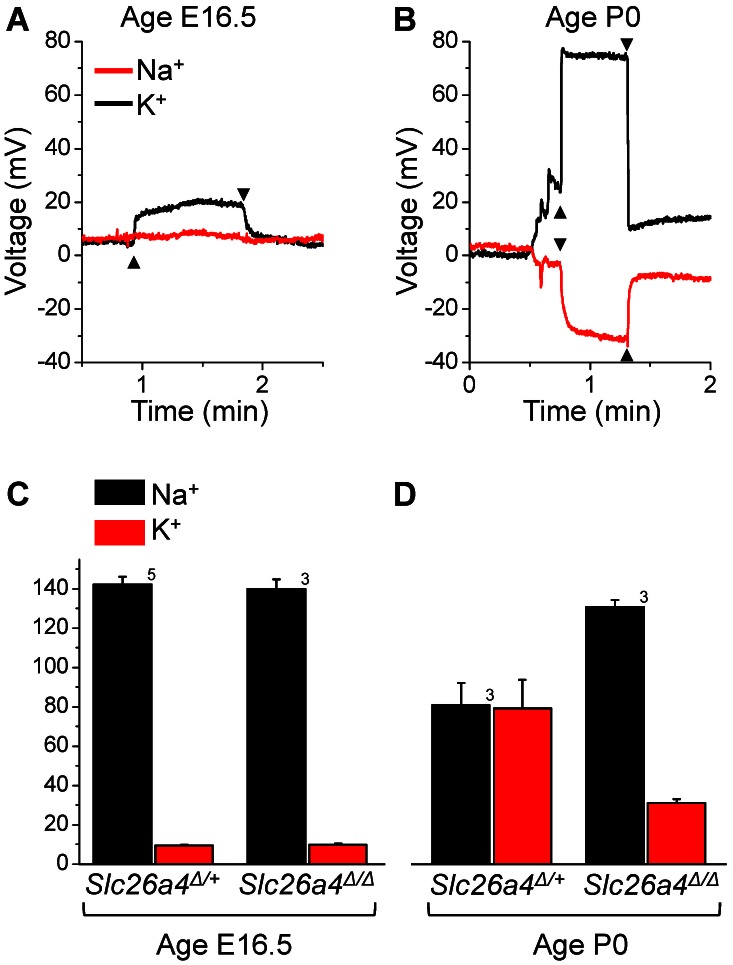
Measurements of the endolymphatic Na^+^ and K^+^ concentrations *in vitro*. **A**) Representative traces of the Na^+^ (*red*) and K^+^ (*black*) concentration in the cochlea of an *Slc26a4* ^Δ/+^ mouse at age E16.5. **B**) Representative traces of the Na^+^ (*red*) and K^+^ (*black*) concentration in the cochlea of an *Slc26a4* ^Δ/+^ mouse at age P0. **C–D**) Summary of Na^+^ and K^+^ concentration measurements in cochlear endolymph in *Slc26a4* ^Δ/+^ and *Slc26a4* ^Δ/Δ^ mice at age E16.5 and P0. Numbers next to bars represent the number of animals.

The *in vitro* method was further used to determine the endolymphatic Na^+^ and K^+^ concentrations in the cochlea of *Slc26a4* ^Δ/+^ and *Slc26a4* ^Δ/Δ^ mice at ages E16.5 and P0 (Fig. 5). At both ages the endolymphatic K^+^ concentration had been found in both genotypes to be significantly higher than the perilymphatic K^+^ concentration and the transepithelial voltage had been found to be zero, *see above*. Measurements were made with double-barreled Na^+^ and K^+^ selective electrodes. Recordings with the K^+^ electrode were used as indicator of a successful penetration into scala media (Fig. 5A and 5B).

At age E16.5, the endolymphatic Na^+^ and K^+^ concentrations in the cochlea were 141±3 (n = 8) mM and 9.6±0.3 (n = 8) mM, respectively. No differences were detected between *Slc26a4* ^Δ/+^ and *Slc26a4* ^Δ/Δ^ mice. At age P0, the endolymphatic Na^+^ dropped in *Slc26a4* ^Δ/+^ mice to 80±12 (n = 3) mM and the endolymphatic K^+^ concentrations rose to 80±14 mM (n = 3). Smaller changes were observed in *Slc26a4* ^Δ/Δ^ mice where the endolymphatic Na^+^ concentration dropped to 131±4 (n = 3) mM and the K^+^ concentration rose to 31±2 (n = 3) mM. No difference was found between the genotypes in the sum of the Na^+^ and the K^+^ concentration (Fig. 5C and D). At E16.5 the sum was 151±3 (n = 8) and at P0 the sum was 160±5 (n = 6). The perilymphatic Na^+^ and K^+^ concentrations did not vary with genotypes or age and were 147±3 (n = 14) mM and 4.2±0.3 (n = 14) mM, respectively. The sum of the perilymphatic Na^+^ and K^+^ concentrations was 151±3 mM (n = 14).

### HCO_3_^−^ and Cl^−^ concentration estimations in the cochlea

The HCO_3_^−^ concentrations in cochlear endolymph were estimated under the assumptions that CO_2_, HCO_3_^−^ and pH are in equilibrium such that the HCO_3_^−^ concentration can be calculated based on measurements of the endolymphatic pH. In the cochlea at age ∼E16.5, the endolymphatic pH had been reported to be pH∼7.44 in *Slc26a4* ^Δ/+^ mice and pH∼7.11 in *Slc26a4* ^Δ/Δ^ mice and the perilymphatic pH had been reported to be pH 7.30 in both genotypes [11]. According to the Henderson-Hasselbalch equation,
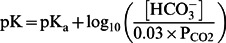



where pK_a_ is the logarithmic acid dissociation constant (pK_a_ = 6.1), ‘0.03’ is the solubility of CO_2_ in water and P_CO2_ is the partial pressure of CO_2_ (P_CO2_ = 5%), the endolymphatic HCO_3_^−^ concentration was calculated to be 25 mM in *Slc26a4* ^Δ/+^ mice and 11.7 mM in *Slc26a4* ^Δ/Δ^ mice and 18 mM in perilymph of both genotypes. With further development, based on endolymphatic pH measurements at P10, the endolymphatic HCO_3_^−^ concentration was calculated to be 35 mM in *Slc26a4* ^Δ/+^ mice and 10.5 mM in *Slc26a4* ^Δ/Δ^ mice [7].

The Cl^−^ concentration in cochlear endolymph was estimated under the assumptions that Na^+^, K^+^, Cl^−^ and HCO_3_^−^ are the major ions in cochlear endolymph, and that the sum of anions matches the sum of cations. Accordingly, the endolymphatic Cl^−^ concentration at age ∼E16.5 was estimated to be 126 mM ( = 151–25) in *Slc26a4* ^Δ/+^ and 139 mM ( = 151–11.7) in *Slc26a4* ^Δ/Δ^ mice and the perilymphatic Cl^−^ concentration was estimated to be 133 mM ( = 151–18) in both genotypes.

### Expression of Atp1a1, Slc12a2, Kcnq1, Scnn1a, Scnn1b and Scnn1g in the cochlea

The molecular mechanism for K^+^ secretion into endolymph has been established in the adult cochlea where K^+^ is taken up across the basolateral membrane of stria marginal cells via the Na^+^/K^+^ ATPase that contains the α-subunit ATP1A1 and the Na^+^/2Cl^−^/K^+^ cotransporter SLC12A2. K^+^ is then secreted into endolymph via the apical K^+^ channel KCNQ1 [23], [24]. Whether this mechanism is responsible for the prenatal rise in the endolymphatic K^+^ concentration is not unknown.

**Figure 6:**
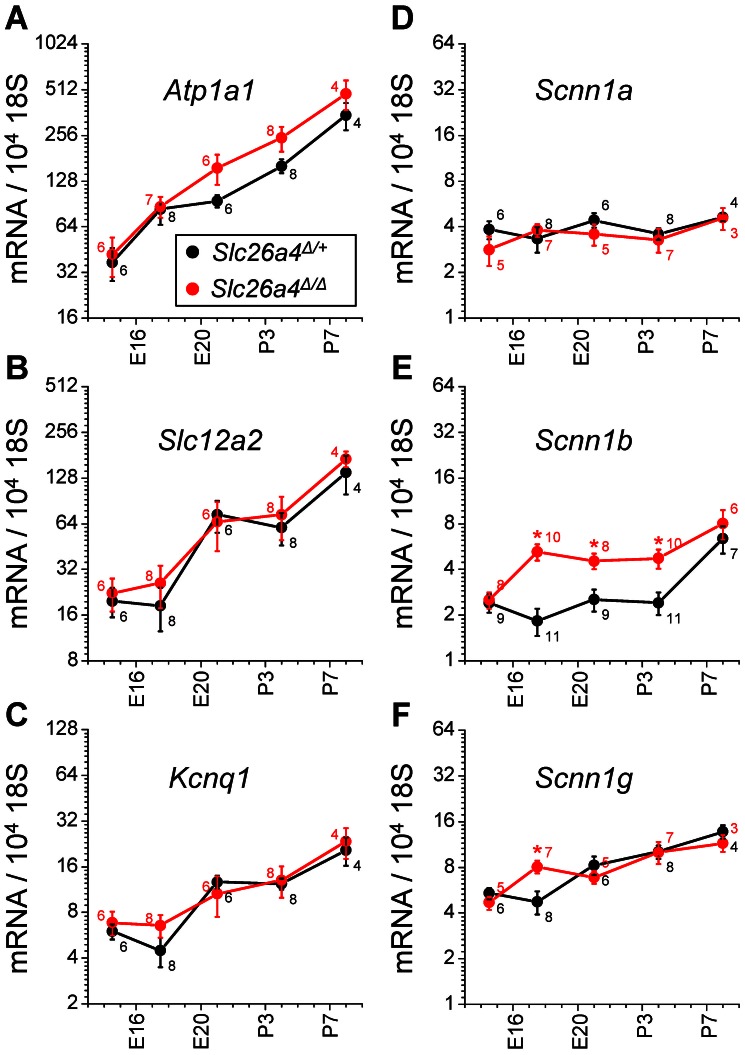
Expression of mRNAs that code for selected Na^+^ and K^+^ channels and transporters in the cochlea of *Slc26a4* ^Δ/+^ and *Slc26a4* ^Δ/Δ^ mice. **A**) The α-subunit of the Na^+^/K^+^ ATPase *Atp1a1*. **B**) The Na^+^/2Cl^−^/K^+^ cotransporter *Slc12a2*. **C**) The α-subunit of the K^+^ channel *Kcnq1*. **D–F**) The α-, β- and γ-subunit of the Na^+^ channel ENaC. Measurements in *Slc26a4* ^Δ/Δ^ mice that differed significantly from measurements in *Slc26a4* ^Δ/+^ mice are marked (*).

Cochlear mRNA expression of *Atp1a1*, *Slc12a2* and *Kcnq1* was quantified between ages E14.5 and P8 and the onset and localization of SLC12A2 and KCNQ1 protein expression was determined in the cochlea of *Slc26a4* ^Δ/+^ and *Slc26a4* ^Δ/Δ^ mice. Expression levels of *Atp1a1* mRNA rose nearly in parallel between age E14.5 and P8 in *Slc26a4* ^Δ/+^ and *Slc26a4* ^Δ/Δ^ mice (Fig. 6A) with the exception that between E17.5 and P0 a steeper rise was observed in *Slc26a4* ^Δ/Δ^ mice compared to *Slc26a4* ^Δ/+^ mice. In contrast, expression levels of *Slc12a2* and *Kcnq1* were stable between E14.5 and E17.5, increased between E17.5 and P0, where stable between P0 and P4 and then increased between P4 and P8 with no differences between genotypes (Fig. 6B and 6C).

**Figure 7:**
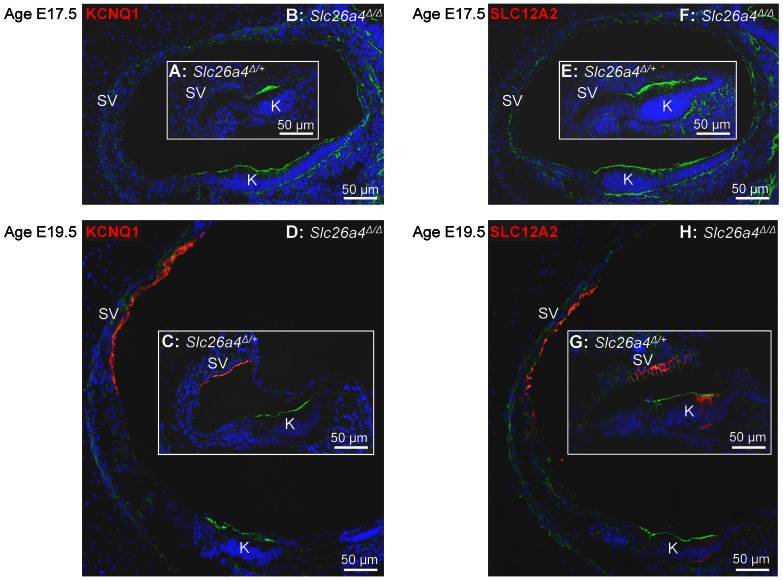
Expression of protein of the K^+^ channel KCNQ1 and the Na^+^/2Cl^−^/K^+^ cotransporter SLC12A2 in the cochlea. **A–D**) Expression of KCNQ1 (*red*) in the cochlea of *Slc26a4* ^Δ/+^ and *Slc26a4* ^Δ/Δ^ mice at ages E17.5 and E19.5. **E–H**) Expression of SLC12A2 (*red*) in the cochlea of *Slc26a4* ^Δ/+^ and *Slc26a4* ^Δ/Δ^ mice at ages E17.5 and E19.5. Note that the onset of expression of KCNQ1 and SLC12A2 occurred in *Slc26a4* ^Δ/+^ and *Slc26a4* ^Δ/Δ^ mice between E17.5 and E19.5. Further, note that images of *Slc26a4* ^Δ/+^ and *Slc26a4* ^Δ/Δ^ mice are reproduced at the same magnification to illustrate the dramatic enlargement in *Slc26a4* ^Δ/Δ^ mice. Stains included DAPI for nuclei (*blue*) and phalloidin for F-actin (*green*). Abbreviations: SV, Stria vascularis; K, Köllikers organ.

Cryosections were prepared from the cochlea of *Slc26a4* ^Δ/+^ and *Slc26a4* ^Δ/Δ^ mice and revealed in *Slc26a4* ^Δ/Δ^ mice the dramatic enlargement of scala media (Fig. 7). At E17.5, no protein expression of SLC12A2 or KCNQ1 was detected, however, expression of both proteins was found at E19.5 in *Slc26a4* ^Δ/+^ and *Slc26a4* ^Δ/Δ^ mice. Expression of SLC12A2 was detected in the basolateral membrane of strial marginal cells and in the basolateral membranes of epithelial cells in Kölliker's organ and expression of KCNQ1 was found in the apical membrane of strial marginal cells. No difference in the location of expression was detected between *Slc26a4* ^Δ/+^ and *Slc26a4* ^Δ/Δ^ mice. These results are based on the evaluation of 2–3 pairs of *Slc26a4* ^Δ/+^ and *Slc26a4* ^Δ/Δ^ littermates that were collected from two timed-pregnant dams per gestational age.

The molecular mechanism for Na^+^ absorption has been established for the adult cochlea in Reissner's membrane epithelial cells as well as in outer sulcus epithelial cells and Claudius' cells [25], [26], [27]. Outer sulcus epithelial cells absorb cations from endolymph including Na^+^ and K^+^, however, Reissner's membrane and Claudius' epithelial cells selectively absorb Na^+^ across the apical membrane via the Na^+^ channel ENaC which consists of the subunits *Scnn1a*, *Scnn1b* and *Scnn1g*. Cochlear mRNA expression of the three ENaC subunits was quantified in *Slc26a4* ^Δ/+^ and *Slc26a4* ^Δ/Δ^ mice between the ages E14.5 and P8. Expression levels of *Scnn1a* were stable during the time of observation and did not differ between *Slc26a4* ^Δ/+^ and *Slc26a4* ^Δ/Δ^ mice (Fig. 6D). In contrast, expression levels of *Scnn1b* rose in *Slc26a4* ^Δ/+^ mice between P4 and P8 and in *Slc26a4* ^Δ/Δ^ mice between E14.5 and E17.5, which is 9–15 days earlier (Fig. 6E). Similarly, expression levels of *Scnn1g* rose between E17.5 and P0 in *Slc26a4* ^Δ/+^ mice and between E14.5 and E17.5 in *Slc26a4* ^Δ/Δ^ mice, which is 1–5 days earlier (Fig. 6F). With the exception of the measurement at E17.5, no difference in expression levels of *Scnn1g* were observed between *Slc26a4* ^Δ/+^ and *Slc26a4* ^Δ/Δ^ mice.

## Discussion

The most salient findings of the present study are that endolymph in the cochlea and the endolymphatic sac of *Slc26a4* ^Δ/+^ and *Slc26a4* ^Δ/Δ^ mice is a NaCl rich fluid during the phase of cochlear growth and enlargement (Figs. 4 and 5), that the onset of the rise in the K^+^ concentration in the cochlea occurs prenatally and coincides with a decline in the Na^+^ concentration (Fig. 5), and that the rise in the K^+^ concentration in the cochlea coincides with the onset of expression of the Na^+^/2Cl^−^/K^+^ cotransporter SLC12A2 and the K^+^ channel KCNQ1 (Fig. 7).

The molecular mechanisms for K^+^ secretion into endolymph have been well established in the adult cochlea, where K^+^ secretion is mediated by marginal cells of stria vascularis [16], [24]. Marginal cells take up K^+^ across the basolateral membrane via the Na^+^/K^+^-ATPase and the Na^+^/2Cl^−^/K^+^-cotransporter SLC12A2 and secrete K^+^ into endolymph across the apical membrane via the K^+^ channel KCNQ1/KCNE1 (formerly called IsK and MinK). The presented data suggest that this mechanism becomes operational with the onset of expression of SLC12A2 and KCNQ1 between E17.5 and E19.5 resulting in a rise of endolymphatic K^+^ concentration in *Slc26a4* ^Δ/+^ and *Slc26a4* ^Δ/Δ^ mice (Figs. 4 and 7). Notably, the onset of the rise in the endolymphatic K^+^ concentration was found to occur prenatally, at E19.5 (Fig. 4A), which is ∼5 days earlier than the previously reported onset at P3 that was based on serially-sectioned freeze-dried labyrinths and X-ray analysis of elemental composition [28], [29]. The expression of SLC12A2 in the cochlea, however, was not limited to strial marginal cells but was also found in the basolateral membrane of epithelial cells in Köllikers organ (Fig. 7). These cells, which are lost during early postnatal development, have previously been shown to express Na^+^/K^+^-ATPase, which suggest they may be engaged in ion secretion [15]. Whether and what ions are transported across the apical membrane is not known.

The molecular mechanism for Na^+^ absorption is well established in Reissner's membrane and Claudius' epithelial cells of the adult cochlea, where Na^+^ absorption from endolymph in mediated via the Na^+^ selective channel ENaC and Na^+^ is extruded across the basolateral membrane via the Na^+^/K^+^-ATPase [25], [27]. Other epithelial cells, such as outer sulcus cells and sensory hair cells, also absorb Na^+^ but do not distinguish between Na^+^ and K^+^ absorption since their major apical cation channel is a cation non-selective channel [26], [30]. The presented quantification of mRNA expression suggest that regulation of Na^+^ absorption, if present, does not occur at the transcriptional level of the channel-forming α-subunit *Scnn1a* or the accessory γ-subunit *Scnn1g*, but occurs possibly at the transcriptional level of the β-subunit *Scnn1b* and at translational or regulatory levels. Interestingly, our observation of upregulation of the β-subunit between P4 and P8 coincides with the onset of amiloride-sensitive Na^+^ transport in Reissner's membrane [31].

The onset of the rise in the K^+^ concentration coincided with the observation of a positive transepithelial potential at E19.5 in *Slc26a4* ^Δ/+^ mice (Fig. 4B). This transepithelial potential may be generated by marginal cells of stria vascularis, which have been shown to generate a positive transepithelial potential when bathed at the apical membrane with a Na^+^-rich solution [24]. This positive potential, however, is different from the endocochlear potential that is generated across the basal cell barrier of stria vascularis, begins to rise at P5, develops within ∼10 days to levels of 90 -100 mV (Fig. 3), and is exquisitely sensitive to anoxia [22]. The positive endocochlear potential is essentially a K^+^ diffusion potential that is generated by the K^+^ channel KCNJ10 that is located in intermediate cells of stria vascularis, which are part of the basal cell barrier [16].

From P3 onward, a negative transepithelial potential was found in the cochlea (Fig. 4B). Negative potentials of similar magnitude have been recorded *in situ* under anoxic conditions [22]. The negative potentials recorded *in vitro* are most likely anoxia potentials that were recorded since our experimental conditions did not maintain normoxic conditions, which are necessary for the positive endocochlear potential. This shortcoming, however, has been shown to not affect the outcome of the ion measurements (Fig. 3C). The negative endocochlear potential found under anoxic conditions (Fig. 3D) is a K^+^ diffusion potential that is thought to be generated by K^+^ channels in the basolateral membrane of the sensory hair cells and that depends on the openness of the non-selective transduction channel in the apical membrane of the sensory cells. Since the present measurements of the transepithelial potential were conducted *in vitro*, which mimics anoxic conditions, the presence of the negative transepithelial potential observed from P3 onward indicates the presence of mature sensory hair cells. This observation is consistent with previous findings on the opening of the transduction channels in murine cochlear hair cells at neonatal stage [32]. The observation that up to P5 neither a positive nor a negative potential was observed in the cochlea of *Slc26a4* ^Δ/Δ^ mice may be related to a retardation of development [12].

The rise in the K^+^ concentration during the neonatal phase of development was found to be delayed by ∼3 days in *Slc26a4* ^Δ/Δ^ mice since K^+^ concentrations reached similar levels at P0 in *Slc26a4* ^Δ/+^ mice and P3 in *Slc26a4* ^Δ/Δ^ mice. This delay in the concentration change may in part be due to the ∼10 fold larger volume of scala media in *Slc26a4* ^Δ/Δ^ mice compared to *Slc26a4* ^Δ/+^ mice that would require a ∼10 fold higher rate of K^+^ secretion and Na^+^ absorption [15]. This delay, however, may also, at least in part, be due to a delayed vascularization of stria vascularis in *Slc26a4* ^Δ/Δ^ mice that had been observed at P3 [11].

The endolymphatic sac has been shown to reabsorb fluid and thereby drain the cochlea during the phase of cochlear lumen formation in *Slc26a4* ^Δ/+^ mice. Failure to drain the cochlea leads to enlargement of the cochlea in *Slc26a4* ^Δ/Δ^ mice [15]. It is conceivable that loss of *Slc26a4* disrupts NaCl absorption and thereby leads to the enlargement. The negative transepithelial potential found in the prenatal endolymphatic sac (Fig. 4D) is consistent with electrogenic Na^+^ absorption mediated by a channel such as ENaC, which has been found to be expressed in the endolymphatic sac [33]. Whether ENaC or other ion transporters mediate fluid absorption in the endolymphatic sac is beyond the scope of the present study.

In summary, we have determined 1) that endolymph in the cochlea and the endolymphatic sac of *Slc26a4* ^Δ/+^ and *Slc26a4* ^Δ/Δ^ mice is a NaCl rich fluid during the phase of cochlear growth and enlargement, 2) that the onset of K^+^ secretion occurs in the cochlea prenatally, and 3) that the rise in the K^+^ concentration coincides with the onset of expression of the K^+^ channel KCNQ1 and the Na^+^/2Cl^−^/K^+^ cotransporter SLC12A2. The determination of the ionic composition of endolymph during the phase of cochlear growth in *Slc26a4* ^Δ/+^ mice and enlargement in *Slc26a4* ^Δ/Δ^ mice presents an important first step toward elucidating mechanisms that cause the enlargement. The elucidation of these mechanisms is important for the identification of drug targets that can be exploited to prevent enlargement and restore normal cochlear development in patients carrying mutations of *SLC26A4*.
